# Oropouche Virus Genome in Semen and Other Body Fluids from Traveler 

**DOI:** 10.3201/eid3101.241452

**Published:** 2025-01

**Authors:** Zsófia Iglói, Widia Soochit, Bas B. Oude Munnink, Adam A. Anas, Karin J. von Eije, Anne van der Linden, Martijn Mandigers, Koen Wijnans, Jolanda Voermans, Felicity D. Chandler, Annemiek A. van der Eijk, Corine GeurtsvanKessel, Richard Molenkamp, Reina S. Sikkema, Babs Verstrepen, Marion Koopmans

**Affiliations:** Erasmus Medical Center, Rotterdam, the Netherlands (Z. Igloi, W. Soochit, B.B. Oude Munnink, A.A. Anas, K.J. von Eije, A. van der Linden, M. Mandigers, K. Wijnans, J. Voermans, F.D. Chandler, A.A. van der Eijk, C. GeurtsvanKessel, R. Molenkamp, R. S. Sikkema, B. Verstrepen, M. Koopmans); World Health Organization Collaborating Centre for Arbovirus and Haemorrhagic Fever Reference and Research, Rotterdam (Z. Igloi, B.B. Oude Munnink, C. GeurtsvanKessel, R. Molenkamp, R.S. Sikkema, B. Verstrepen, M. Koopmans)

**Keywords:** *Suggested citation for this article*: Igloi Z, Soochit W, Oude Munnink BB, Anas AA, von Eije KJ, van der Linden A, et al. Oropouche virus genome in semen and other body fluids from traveler. Emerg Infect Dis. 2025 Jan [*date cited*]. https://doi.org/10.3201/eid3101.24241452

**To the Editor:** We read with interest the article by Castilletti et al. that showed prolonged shedding of Oropouche virus (OROV) in various body fluids (*1*). In addition, the authors isolated OROV from semen of 1 patient. The findings of that report coincide with multiple relevant findings, including our similar observation and findings of OROV-specific IgM in 6 of 68 newborns with microcephaly (*2*) and of OROV vertical transmission resulting in fetal death (*3*).

Using real-time reverse transcription PCR (RT-PCR), we detected OROV infection in a male patient returning to the Netherlands from Cuba in August 2024 (*4*). We also detected OROV seroconversion by using in-house methods and excluded dengue virus infection. The patient recovered without complications and agreed to donate follow-up samples (i.e., urine, blood, feces, and semen). OROV genome was still detectable by real-time RT-PCR in all samples except feces up to 32 days after symptom onset, which is longer than was generally described (*5*) before the article published by Castilletti et al. (*1*). RT-PCR cycle threshold (Ct) values in all specimens gradually increased, and thus viral load reduced over time. Urine and semen samples obtained 17 and 32 days after symptom onset had the lowest Ct values (Ct 21.8 for urine and 25.5 for semen on day 17, and 24.7 for urine and 29.8 for semen on day 32), but virus culture was unsuccessful. We obtained near full-length genomes from serum, urine, and semen at both time points by using amplicon-based Nanopore sequencing (*6*) (GenBank accession nos. PQ572768–PQ572779). Moreover, the partial sequence obtained from the later semen sample contained 2 single-nucleotide polymorphisms in the large segment, which may indicate prolonged virus replication in the immune-privileged testis.

The increasing evidence that OROV infection during pregnancy can affect fetal development is concerning. Although sexual transmission of OROV has yet to be fully studied, our findings, along with those of Castilletti et al., indicate potential. However, the outbreak, although slowing, is still ongoing in Central and South America.

## References

[1] Castilletti C, Huits R, Mantovani RP, Accordini S, Alladio F, Gobbi F. Replication-competent Oropouche virus in semen of traveler returning to Italy from Cuba, 2024. Emerg Infect Dis. 2024;30:2684–6. 10.3201/eid3012.241470 39374598 PMC11616654

[2] das Neves Martins FE, Chiang JO, Nunes BTD, Ribeiro BFR, Martins LC, Casseb LMN, et al. Newborns with microcephaly in Brazil and potential vertical transmission of Oropouche virus: a case series. Lancet Infect Dis. 2024;S1473-3099(24)00617-0. 10.1016/S1473-3099(24)00617-0 39423837

[3] Garcia Filho C, Lima Neto AS, Maia AMPC, da Silva LOR, Cavalcante RDC, Monteiro HDS, et al. A case of vertical transmission of Oropouche virus in Brazil. N Engl J Med. N Engl J Med. 2024;•••:c2412812. 10.1056/NEJMc2412812 39476360

[4] Naveca FG, Nascimento VAD, Souza VC, Nunes BTD, Rodrigues DSG, Vasconcelos PFDC. Multiplexed reverse transcription real-time polymerase chain reaction for simultaneous detection of Mayaro, Oropouche, and Oropouche-like viruses. Mem Inst Oswaldo Cruz. 2017;112:510–3. 10.1590/0074-02760160062 28591313 PMC5452489

[5] Cardoso BF, Serra OP, Heinen LB, Zuchi N, Souza VC, Naveca FG, et al. Detection of Oropouche virus segment S in patients and in*Culex quinquefasciatus* in the state of Mato Grosso, Brazil. Mem Inst Oswaldo Cruz. 2015;110:745–54. 10.1590/0074-02760150123 26517653 PMC4667577

[6] Naveca FG, Almeida TAP, Souza V, Nascimento V, Silva D, Nascimento F, et al. Human outbreaks of a novel reassortant Oropouche virus in the Brazilian Amazon region. Nat Med. 2024. 10.1038/s41591-024-03300-3 39293488

